# Report on Vaccination

**Published:** 1864-05

**Authors:** Swayne Wickersham

**Affiliations:** Chicago, Ill.


### CHICAGO MEDICAL EXAMINER.

N. S. DAVIS, M.D., Editor.

VOL. V. MAY, 1864. NO. 5.

### Original Contributions.

ARTICLE XIX.

### REPORT ON VACCINATION.

By SWAYNE WICKERSHAM, M.D., Chicago, Ill.

Communicated to the Chicago Medical Society.

Mr. President and Gentlemen:—Your Committee, to whom was referred the subject of vaccination, having had the same under careful consideration, beg leave to submit the following report:—

In order to duly appreciate the great boon conferred upon the human family by the introduction of vaccination, we must bear in remembrance the great ravages of small-pox previous to the discovery of the preventive.

It is deemed proper to incorporate in this report a very brief history of variola. The period at which small-pox was first observed, is not definitely known. It may have been coincident with the birth of man. It may not have appeared until long after the origin of the human race. That it was of very ancient origin, we have an abundance of historical testimony. I believe there is no recorded history which warrants us in saying that it has had a longer existence in any part of the world than in India and China, where their histories claim that it has prevailed from time immemorial. It is said to have shown itself in Arabia about the time of the birth of Mohammed, and to have subsequently invaded Syria, Egypt, and Southern Europe, with the armies of his successors.

We are destitute of any satisfactory proof that it was known to either the ancient Greeks or Romans. The mortality from the disease, always great, was at times terrible. Nothing so positively demonstrates the progress of medical science as to compare the means at our disposal to check its ravages, with those at the command of physicians, previous to the discovery of vaccination, by the immortal Jenner. So large a proportion of the cases of small-pox in our day present themselves in a modified form, occurring in persons who have at a former period been vaccinated, that the average mortality has been greatly reduced. We also believe that improved methods of treatment have been productive of similar though less marked results.

To such an extent have the horrors of variola been mitigated, that the public are exhibiting signs of apathy, and are to a considerable degree neglecting to avail themselves of those means, which alone have stripped small-pox, of those terrible consequences which formerly attended it. We propose to insert some mortuary statistics in order to illustrate the natural history of the disease, in communities where, or in persons in whom, it has not been influenced or modified by previous vaccination.

Prof. Hewitt, of London, associated with the St. Mary's Hospital Medical School, says that, out of every 100 persons attacked, about 35 die.

In the case of children under five years of age, out of every 100 attacked, 50 will die. From the age of five to ten, out of every 100 attacked, 27 will die; from ten to fifteen, 23 will die; from fifteen to twenty, 26 will die; from twenty to twenty-five, 40 will die; from twenty-five to thirty, 45 per cent. will die; from thirty to thirty-five, 57 per cent.; from forty to sixty, 69 per cent.; and between sixty and ninety, the mortality is 75 per cent. It will be observed that the mortality is the largest among the very young and old. Of 2,654 patients admitted, during sixteen years, into the London Small-pox Hospital, affected with the disease and unvaccinated, 996 died, which is 37 per cent.

In Sweden, in the twenty-eight years before the introduction of vaccination, the deaths from small-pox were 2,050 per annum for every million of the population. Forty years afterwards, they were but 158 per annum for every million of the people. In Westphalia, between 1776 and 1780, the death-rate from small-pox was 2,643 per annum for every million inhabitants; but, after vaccination was well established, it was but 114. In Copenhagen, for the last fifty years of the last century, the deaths were 3,128 per annum for every million of persons; but the first fifty years of the current century gave but 286 per annum for the same number of the population.

The above mortality is almost inconceivable, yet nevertheless true. The modified form of the disease is attended with much less mortality, the per centage depending upon the goodness and efficiency of the vaccination. The above statistical observations do not fully demonstrate the deplorable consequences of the malady. A very large number of those who are permitted to recover, do so at the expense of beauty and former health. Not only does small-pox kill its 35 per cent. of those whom it attacks, but the disfigurement by scars, loss of vision, permanently shattered health, make the disease doubly unwelcome.

Many a maiden, formerly noted for her beauty,—many a manly youth, formerly full of ambition, industry, and talent, accustomed to indulge in high aspirations,—arises from a bed of small-pox to behold themselves again, not as formerly, but now possessed for all the future, of hideous and repulsive countenances. In many instances, such unfortunates have yearned for death, and sought and obtained the grave of a suicide. In consideration of the above, it is not wonderful that physicians from an early period exercised their ingenuity to obtain some means by which they would be enabled to ameliorate the consequences of the disease. But little progress was made in that direction until the discovery of inoculation. When, where, and by whom inoculation was first performed, is involved in much obscurity, and perhaps will never be positively ascertained.

As a means of rendering small-pox milder and more tractable, the inoculation of the disease was practiced for a long period by some of the nations of Asia, before its benefits were known to Europeans. It was practiced in Constantinople about 1700; it was introduced into England early in the year 1721, by the celebrated Lady Mary Wortley Montague, whose husband had been an English ambassador at Constantinople. In June, 1721, it was introduced into America by the Rev. Cotton Mather, under the direction of Dr. Boylston, of Boston. It was performed in different ways, but mostly in a manner similar to that to which we resort in performing vaccination. It was unquestionably, after a brief period had elapsed, regarded with much favor. It succeeded in disarming the disease of some of its most frightful features, and sensibly diminished its mortality; but its benefits were not unadulterated, notwithstanding its advantages outweighed its disadvantages: there were objections to its use. It would probably do good—possibly harm. It multiplied the *foci* of contagion; had, in consequence, a tendency to perpetuate small-pox and cause it to frequently prevail as an epidemic, while previously long intervals often elapsed during its absence. Occasionally death resulted, although Gregory says that, if carefully managed, not more than one death would occur in five hundred cases. We believe this estimate too small.

Happily Jenner comes to our aid in 1798, and, by his invaluable discovery, renders entirely unnecessary the further performance of the operation. Subsequent to Jenner's discovery, laws were passed in many of the American States, prohibiting inoculation. Pennsylvania passed such a law in 1811, attaching a heavy penalty for its violation. It was also prohibited by law in Great Britain, though we believe not until the year 1840. Asia and Africa, the most ancient seats of small-pox, still avail themselves of this mode of palliation.

We would not have said even thus much about inoculation had it not have been for an effort that had been made by an eminent physician and scholar to revive it. I allude to Prof. Dickson, of Philadelphia, who occupies the Chair of Practice of Medicine in the Jefferson College. In the July number of the American Medical Journal for 1862, he publishes a very able article in advocacy of its revival. It seems that he was induced to publish such a recommendation from the facts embraced in the annual report on Meteorology and Epidemics for 1861, read by Dr. Jewell to the Philadelphia College of Physicians. There was, during a part of the year 1861, a general prevalence of the exanthemata in Philadelphia, resulting in a very heavy mortality, especially from small-pox. There died in that city, during the year 1861, 758 persons from small-pox, which was twice as great a number as had died in any year except 1852, during the current century.

He seems to have entertained the idea that vaccination was a partial failure, and the deficiency should be supplied by inoculation. He says "we cannot avoid being shocked at this retrograde exhibition and shrinking from the acknowledgment of professional failure and defeat which it seems to imply." He recommends a combination of the two, "inoculation, superimposed upon vaccination." Prof. Dickson could not have had in his mind Doctor Woodville's evidence, given before a commission appointed by the British Parliament to inquire into the subject in 1802, who says "that within the two years, 1799 to 1801, 7,500 persons were vaccinated at the Small-pox Hospital, of whom about one-half were subsequently inoculated with small-pox matter, and in none of them did small-pox produce any effect. Though no doubt the result would have been different had the inoculation been deferred for some years. We know of no experiments of this nature that have been performed recently, except those of Dr. Darrach, of Philadelphia, who inoculated three of his children with small-pox matter who had been vaccinated in infancy. The children were aged 12, 13, and 14. In two there was a slight local affection, without any fever or eruption. In the third case, there was local affection without fever, but with papular eruption on the seventh day, not advancing to vesicles.

As this subject is one of such vast importance, we will allow the eminent Professor to speak for himself. He says, "Indeed, so much are the violence, the suffering, and the proportional mortality of small-pox diminished by inoculation, that I would advocate unhesitatingly the propriety of universal inoculation at as early a period of life as was ascertained by repeated experiment to be safe and allowable. I would have such inoculations repeated at short intervals, until in every subject the point of absolute incapacity to receive the affection was fairly reached. This would happen in the majority of instances with the first efficient insertion of the virus. Those who had gone through this process would be proof for the future against the pestilence; and, as to them, it would be annihilated—virtually exterminated. And as all constitutional peculiarities are hereditarily transmitted, whether of original organization or in any way acquired, so this antiproclivity or acquired immunity would go down increasing in force with every generation, until the whole race would become insusceptible of this horrid mode of dying. Let us procure that it shall be ordained that every child shall undergo vaccination by some expert within a month after birth; that, as soon as the constitution shall have gone through its influence, inoculation with variolus matter shall be performed, and repeated at brief intervals."

We regard all this, although uttered by an experienced and erudite man, as mere utopian speculation. It would be almost impossible to carry into effect such a plan, even though it were advisable. Neither is it proven that persons so treated would be any more thoroughly protected than those who would have performed upon them the same number of vaccination repetitions. We had under our care this winter quite a severe case of varioloid in a woman who had in youth-time been inoculated. She insisted, previous to the attack, that she was safe, although hourly exposed to the infection of variola. In Davy's tables, 97 are set down as having had small-pox previously. He mentions as authentic the case of a woman, mother of ten children, who had small-pox eleven times! first in infancy, and afterwards when each of her children had it, and the last attack as severe as the first. If medical testimony can be relied upon, there have been many similar instances. No intelligent physician calls in question the fact that a person may have a second attack of the disease, and that a second attack is not always life protective. The same difficulties will attend re-inoculations that attend vaccinations; persons will be equally neglectful under either system. We are opposed to the retrograde movement, and do not favor the reintroduction of inoculation, but insist that we have in vaccination a safe and certain preventive, not simply a palliative or modifier, as some choose to call it; but I firmly believe that we must handle it differently from what we do. We must obtain from it all the power it possesses, if we would exterminate the loathsome and pestilential affection. We will now briefly glance at the history of vaccination, after which we will express our views as to the manner in which it should be used, if we would accomplish all that we desire. That a disease sometimes appeared in the cow, which, if imparted to man, secured him against small-pox, was known long anterior to the time of Jenner. This fact was acted upon to a limited extent in different parts of the world, as in India, Persia, and some parts of South America. In some of the dairy counties of England, it was long since observed that the cows were subject to a pustular eruption, and the popular belief was, that if this disease was communicated to man, that he was safe from the influence of small-pox. To this circumstance, Dr. Edward Jenner directed his attention early in life. He was not, as is ordinarily supposed, the first to practice the artificial communication of the vaccine disease. But no one shares with him the credit of having brought the art into almost universal use. The inestimable advantages which have accrued to the human family, have immortalized him. He met with violent opposition, but we are truly thankful that his ardent enthusiasm, amidst all his discouragements, was not extinguished. The British Parliament recognised his claim and voted him £30,000 sterling.

It was in 1796, that Dr. Jenner made his first successful experiment. He was then residing in Gloucester county, in England, largely engaged in inoculation. He ascertained that those in whom the matter from the cow was introduced, were insusceptible to inoculation. His first vaccination was per formed in 1796, upon a boy aged 8 years. In 1798, he published his investigations in an essay, entitled "Inquiry into the causes and effects of variola, vaccinal." In this paper he set forth his proofs so prominently that they attracted immediate attention and soon enlisted a host of co-operators. His assertions were verified. In 1799, it was introduced into the United States, and in 1800, into France and other parts of continental Europe. Jenner, in his first essay, announced as his conviction that when cow-pox had passed in a perfect manner through the system, that it was secure from the infection of small-pox forever. This opinion was mutually entertained by most of the physicians in Europe and America, until 1818. During that year an epidemic of small-pox pervaded Scotland; and many of those who had been vaccinated were its victims. Soon after, the same was observed in other localities. These facts stimulated physicians to make a more close investigation. The confidence of some was shaken in its prophylactic value, and I am sorry to confess that there are those among us today, prominent in the profession, who advise a return to inoculation. We have already stated that we are not of that number. Many great and good men have entertained the happy thought that vaccination was destined to make small-pox a disease of the past.

That future generations should have no personal knowledge of it, but, in order to accomplish this much-desired object, we must have universal vaccination and universal re-vaccination. Unless compulsory laws are passed, this will not be done. It must not be left optional with the people, a mere voluntary act. There will be in every community those who will be negligent and apathetic; others who will doubt its prophylactic virtues and discourage the operation. The United States, in this particular, are far behind many of the European nations. Many of the latter have laws requiring the operation, and they are not violated with impunity. In such communities, small-pox is almost unknown, except when carried into them by strangers. An Act of Parliament went into force in Ireland, on the 1st of January of this year, making it compulsory that every child, born after that date, should be vaccinated within six months. For non-compliance with this regulation, parents and guardians are liable to a fine of ten shillings. We sincerely hope that this law will be enforced, and that, in due season, we will not witness so large a number of the noble and toil-worn sons and daughters of the Emerald Isle come to us with such disfigured countenances. We think the above law is somewhat objectionable for not making the limit three months instead of six, and not providing for re-vaccination. Dr. Jewell, of Philadelphia, says, in one of his annual reports, "That he desires to impress upon the Profession the inadequacy of voluntary provision to secure us from the ravages of small-pox. And further, that nothing less than a compulsory law, with a penalty attached for its violation, will prove an effectual barrier." Another eminent writer says, that "the absence of an efficient system of protection should be stigmatized as a national disgrace—almost a national crime." Another, equally eminent, says, "Believing, however, as I do, that the introduction and propagation of this pestilence are well enough understood for all practical purposes, I do regard all governments as responsible for the institution of proper and relevant efforts at prevention and circumscription, and deeply guilty when this is neglected." These are our views, with a certain degree of limitation. Guilt implies knowledge. If a government is ignorant, and acts prejudicially to the welfare of the people, we question the guilt. But if fully informed, and then acts in opposition to the best interests of its constituents, none can doubt the guilt. We believe there is no compulsory law in this city bearing upon vaccination. We assume that our municipal government is ignorant of its necessity. We think that it behoves this Medical Society, as the representatives of the Profession in this city, to inform them of the importance of such a law. That it will then be their duty to pass such an ordinance, and also to see that it is enforced. Neither should they rely upon our noble and philanthropic Profession to vaccinate gratuitously all who are unable to pay. We recommend that this Society shall advise them to appoint vaccinators for the poor, at a reasonable annual salary. Also, that they shall prohibit any vaccinations, except performed by experts; that the public vaccinator shall be required to assist in a rigid enforcement of the law; that every child, born or brought to this city, shall be vaccinated within a limited period of time, and that a heavy penalty shall be impartially imposed for every violation of said law. If such a law had have been in existence in this city, and enforced even one year ago, what a world of suffering would have been saved! The lives of a large number of infants and children would have been preserved to have taken their places in the great and busy hive of the world. Many of the poorer classes feel their inability to recompense a physician who is qualified to vaccinate. They are compelled to resort to illiterate quacks who are not capable of judging as to the protective power of the so-called vaccinations; and who, placing but a small valuation upon their services, charge but a mere pittance. During last August, we witnessed, in the North Division, on Larrabee and Kingsbury streets, some of the deplorable consequences of the above. Within a small circuit there were many cases of small-pox and several deaths, among very young children. We were informed that they had no confidence in the protective power of vaccination. They based their convictions upon the fact that some four children, who had been vaccinated the spring previously, had contracted variola, and in whom it was said the vaccinations had been successful. Confident that they were entertaining erroneous impressions, we took considerable pains to dispel the error. Two of the children referred to were dead and buried, and whom I did not see. One was then in *articulo-mortis.* There was no vaccine cicatrix upon either of the children then living. The people were very illiterate and supposed the vaccination had taken because the children had an eruption subsequently upon their persons. The occurrence of small-pox in these cases had deterred others from protecting their children by vaccinations, laboring under the impression that it was of no utility. The operation was performed by one with whom we would refuse association, and who in my opinion was incapable of discriminating between good and bad vaccination. I also attended, last autumn, three German children suffering with small-pox, on South Wells street, between Harrison and Polk. These children had been vaccinated a few months previously, by an ignorant German woman. There was no evidence that the vaccination had been successful. There were no vaccine cicatrices. There was an abundance of negative testimony. She asserted very positively that those three children, one of whom succumbed from the small-pox, were as well vaccinated as any of the three hundred that she had done during the year. Many of you remember the quack Doctor of this city, who a few years ago purchased some matter at one of our stores, and who in his ignorance made use of the envelope of the matter, instead of the matter itself. It is needless to say that his cases were still susceptible to small-pox. These circumstances might be multiplied, but they are sufficient to demonstrate the necessity of our municipal government coming to the aid of the people and appointing public vaccinators, selecting capable men, and prohibiting the performance of the operation by the mere pretender. I believe, in most of the Atlantic cities, that there are physicians appointed to attend to the vaccination of the poor. If eternal vigilance is the price of liberty, so is eternal vigilance the price of exemption from small-pox. We know that we are not deriving the benefit from vaccination that we should. The forceps in obstetrics, Peruvian bark in malarial fevers, anæsthetics in surgery, were most valuable discoveries, but combine the benefits of all, and they do not equal that which has resulted from vaccination. We further believe that even among those of us who claim to be educated, and in possession of all the requisite information, can be found many who are not performing the operation in a manner calculated to give our patients the greatest amount of preventive security.

We do not wish to extend this report, but we feel that we cannot close it until we incorporate in it our objections to the common method adopted by the American physician in vaccinating. The majority of us trust to the introduction of matter in a single place, producing one cicatrix. If it is successful, we concede its efficiency for a limited period of time. Some will be protected for life. But we are well satisfied, from many observations, that the protective power of vaccinations is, as a rule, greatly extended; that it secures a much larger number for life-time; and, if subsequently attacked with small-pox, much less likely to die, if there have been at the original vaccination three, four or more insertions of the matter in different parts of the body. Perhaps the same security could be purchased by re-vaccinations, but we all know the proneness of people to neglect them; hence we think it our duty to give them, while under our care, the greatest security of which vaccination is capable. The statistical tables, that have been recently published in London, give us additional assurance that we are correct. I quote from Mr. Marson's very valuable observation. He says, "that of the whole number of individuals attacked with small-pox, and previously vaccinated, seven per cent. die; of the badly-vaccinated, fifteen per cent. die; but in well-vaccinated persons—understanding by the term "well vaccinated" those having four or more cicatrices—the mortality is less than *one per cent.* He finally states, in reference to the statistics, "Test the question in which way soever you will, the result is in favor of producing four vesicles, at least, at vaccination.

In the public institutions of London, there are annually vaccinated about 20,000 individuals, and the class of persons all will admit, if subsequently attacked with small-pox, would go to the Hospital. But we are told that only about nine cases per annum of those publicly vaccinated, are admitted as small-pox patients, and that there has been but one death out of 36,000 of such patients. We believe that all the public vaccinators in London introduce the matter in several places. Prof. Hewitt, of London, says, "The power of vaccination to prevent those attacked with small-pox, from dying of the disease, is thus in direct ratio to the goodness and efficiency of vaccination." He urges four or more insertions.

We recommend an immediate abandonment of the common mode of vaccinating, and that hereafter we shall trust to no less than four insertions at a vaccination. There are many other points to which we would like to allude, such as the selection and preservation of the material, but our report is already much too lengthy.

We ardently hope that our recommendations to the Society, that it exercise its influence to obtain the enactment of laws for compulsory vaccination, will be carefully considered. In conclusion, I desire to say that I do not know that this report embodies the views of the other members of the Committee. I am responsible for all its contents.

All of which is most respectfully submitted.

